# Pharmacological characterisation of the interaction between glycopyrronium bromide and indacaterol fumarate in human isolated bronchi, small airways and bronchial epithelial cells

**DOI:** 10.1186/s12931-016-0386-8

**Published:** 2016-06-13

**Authors:** Mario Cazzola, Luigino Calzetta, Ermanno Puxeddu, Josuel Ora, Francesco Facciolo, Paola Rogliani, Maria Gabriella Matera

**Affiliations:** Department of Systems Medicine, Chair of Respiratory Medicine, University of Rome Tor Vergata, Rome, Italy; Department of Systems Medicine, Respiratory Pharmacology Research Unit, University of Rome Tor Vergata, Via Montpellier 1, 00133 Rome, Italy; Division of Respiratory Medicine, University Hospital Tor Vergata, Rome, Italy; Regina Elena National Cancer Institute, Thoracic Surgery Unit, Rome, Italy; Department of Experimental Medicine, Unit of Pharmacology, Second University of Naples, Naples, Italy

**Keywords:** Glycopyrronium bromide, Indacaterol fumarate, Synergistic interaction, Human bronchi

## Abstract

**Background:**

Nowadays, there is a considerable gap in knowledge concerning the mechanism(s) by which long-acting β_2_-agonists (LABAs) and long-acting muscarinic antagonists (LAMAs) interact to induce bronchodilation. This study aimed to characterise the pharmacological interaction between glycopyrronium bromide and indacaterol fumarate and to identify the mechanism(s) leading to the bronchorelaxant effect of this interaction.

**Methods:**

The effects of glycopyrronium plus indacaterol on the contractile tone of medium and small human isolated bronchi were evaluated, and acetylcholine and cAMP concentrations were quantified. The interaction was assessed by Bliss Independence approach.

**Results:**

Glycopyrronium plus indacaterol synergistically inhibited the bronchial tone (medium bronchi, +32.51 % ± 7.86 %; small bronchi, +28.46 % ± 5.35 %; *P* < 0.05 vs. additive effect). The maximal effect was reached 140 min post-administration. A significant (*P* < 0.05) synergistic effect was observed during 9 h post-administration on the cholinergic tone, but not on the histaminergic contractility. Co-administration of glycopyrronium and indacaterol reduced the release of acetylcholine from the epithelium but not from bronchi, and enhanced cAMP levels in bronchi and epithelial cells (*P* < 0.05 vs. control), an effect that was inhibited by the selective KCa^++^ channel blocker iberiotoxin. The role of cAMP-dependent pathway was confirmed by the synergistic effect elicited by the adenylate cyclase activator forskolin on glycopyrronium (*P* < 0.05 vs. additive effect), but not on indacaterol (*P* > 0.05 vs. additive effect), with regard of the bronchial relaxant response and cAMP increase.

**Conclusions:**

Glycopyrronium/indacaterol co-administration leads to a synergistic improvement of bronchodilation by increasing cAMP concentrations in both airway smooth muscle and bronchial epithelium, and by decreasing acetylcholine release from the epithelium.

## Background

Treatment for patients suffering from chronic obstructive pulmonary disease (COPD) not controlled by a single bronchodilator requires the addition of a second bronchodilator characterised by a different mechanism of action [1]. We strongly support this therapeutic approach because using multiple drugs in combination may allow lower doses of individual agents, decrease adverse effects, simplify medication regimens and improve compliance [2].

To date, there is solid clinical information for combining β_2_-adrenoceptor agonists and anti-muscarinic agents [3–5], and recently, the true nature of the pharmacological interaction between long-acting β_2_-agonists (LABAs) and long-acting anti-muscarinic antagonists (LAMAs) was elucidated in both *ex vivo* studies performed in human isolated airways and clinical trials in COPD patients [6–8]. Indeed, it is now clear that combining low concentrations of a LABA with a LAMA leads to synergistic relaxation of human airway smooth muscle (ASM), which, in turn, provides optimised effectiveness while reducing the risk of side effects [9].

Although the synergistic interaction between the LAMA aclidinium bromide and the LABA formoterol fumarate has been deeply characterised from a pharmacological point of view [6], we cannot exclude the possibility that different LABA/LAMA combinations such as glycopyrronium bromide (NVA237) plus indacaterol fumarate (QAB149) may show a different pharmacological interaction [10].

Moreover, although several pathways have been proposed to clarify the intracellular cross-talk elicited by combining β_2_-adrenoceptor agonists and anti-muscarinic agents in ASM cells and parasympathetic neurons [9, 11], there is still a considerable gap in knowledge with regard to the pharmacological mechanism(s) by which a LABA and a LAMA interact when they induce bronchodilation.

Therefore, this study aimed to characterise the nature (additive or synergistic) of the interaction between glycopyrronium and indacaterol in human isolated bronchi and bronchioles and to identify the mechanism(s) leading to a bronchorelaxant effect due to such an interaction.

## Methods

### Ethical approval and informed consent

Ethical approval and informed consent were obtained from the University of Rome ‘Tor Vergata’ (R.S. 107.14/2014, Rome, Italy), and were consistent with the guidelines of the 2009 National Committee of Bioethics, the recommendations of the National Committee of Bio-safety, Biotechnology and Sciences (Italy) on the collection of biologic samples for research purposes, the 2010 Italian ethical and legal recommendations concerning biobanks and research biorepositories (Istituto Nazionale dei Tumori – Independent Ethics Committee, 2010) and the Comitato Nazionale per la Biosicurezza, le Biotecnologie e le Scienze per la Vita (Raccolta di campioni biologici a fini di ricerca, consenso informato, 2009; available at: http://www.governo.it/bioetica/gruppo_misto/Consenso_Informato_allegato_Petrini_2009.pdf).

### Human bronchial tissues

#### Tissue preparation

Macroscopically normal airways were obtained from 23 patients (13 male and 10 female; aged 63.2 ± 2.2 years) undergoing surgery for lung cancer, without a history of chronic airway disease. Detailed demographic characteristics of patients, including smoking history, are reported in Table 1. Samples were taken from areas as distant as possible from the malignancy. Tissues were placed in Krebs–Henseleit (KH) buffer solution (NaCl, 119.0 mmol; KCl, 5.4 mmol; CaCl_2_, 2.5 mmol; KH_2_PO_4_, 1.2 mmol; MgSO_4_, 1.2 mmol; NaHCO_3_, 25.0 mmol and glucose, 11.7 mmol; pH 7.4) containing indomethacin (5 μM) and transported to the laboratory. None of the patients received treatment with xanthines, β_2_-adrenoceptor agonists, glucocorticosteroids or muscarinic antagonists. Preoperative lung function parameters were generally normal, and there were no signs of respiratory infections [12, 13].

**Table 1:** Demographic characteristics of human subjects

Characteristics	Value
Gender (male/female)	13/10
Age (years)	63.2 ± 2.2
Smoking status:	
Current	14
Former	9
Pack years	45.5 ± 8.4
FEV_1_ (L)	2.54 ± 0.14
FEV_1_ (% predicted)	93.15 ± 3.24
FEV_1_ reversibility (%)	3.73 ± 1.68
FVC (L)	3.38 ± 0.17
FEV_1_/FVC (%)	75.23 ± 2.06

Data are expressed as mean ± SEM

#### Isolated bronchi

Airways studied in an isolated organ bath system were cut into rings (thickness: 1-2 mm; diameter: 4-6 mm) and transferred into a 10-mL High Tech 8 Channels Manual Compact Organ Bath system (Panlab Harvard Apparatus, Spain) containing KH buffer (37 °C) and aerated with O_2_/CO_2_ (95 %:5 %). Tissues were allowed to equilibrate, and the KH buffer was constantly changed [12, 13].

#### Epithelium removal

In some experiments, the bronchial epithelium was mechanically removed by using a cotton-tipped applicator gently rubbed for 5 sec on the luminal surface. It has been previously demonstrated that this manipulation does not penetrate the basal membrane and that the lamina propria remains almost intact [14].

#### Videomorphometry

Airways studied using videomorphometry were cut into precision cut lung slices (PCLS) (thickness: <500 μm, diameter: 0.65 ± 0.06 mm) by a Motorised Advance Vibroslice equipped with ceramic blades (Campden Instruments, UK) and then mounted into a visual imaging and patching chamber connected to a Proportional Integral Derivative Temperature Controller with dual thermistor feedback CI7800 (Campden Instruments, UK), containing KH buffer (37 °C) aerated with O_2_/CO_2_ (95 %:5 %). Tissues were allowed to equilibrate, and the KH buffer was constantly changed [6, 15].

### Human bronchial epithelial cells

Primary human bronchial epithelial cells were harvested by gently scraping the luminal airway surface with a convex scalpel blade #10, a procedure that does not penetrate the basal membrane. Collected epithelial cells were pooled in phosphate-buffered saline (PBS) and centrifuged at 500 *g* for 5 min at 4 °C. Bronchial epithelial cells were resuspended, cultured with 1:1 mixture of LHC-9 and RPMI 1640 medium in a volume of 10^6^ cells/mL and maintained at 37 °C in a 5 % CO_2_ humidified incubator [14, 16].

### Contraction measurement

#### Preparation of isolated bronchi and tissue vitality

Bronchial rings were connected to isometric force transducers Fort25 (WPI, UK). The signal was amplified by PowerLab 8/36 and Octal Bridge Amp system (ADInstruments, UK), recorded and analysed using the LabChart 7 interface software (ADInstruments, UK). Tissues were mounted on hooks and attached with a thread to a stationary rod and the other end was tied with a thread to an isometric force displacement transducer. Airways were allowed to equilibrate by flushing with fresh KH buffer solution. Passive tension was determined by gentle stretching of tissue (0.5-1.0 g) during equilibration. The isometric change in tension was measured by the transducer. The tissue vitality and maximal contractile responsiveness was assessed by acetylcholine at a 100 μM concentration and/or by transmural stimulation (also called electrical field stimulation [EFS]) at 25 Hz. These procedures allowed the bronchial rings to be correctly positioned between the hooks. When the response reached a plateau, the rings were washed thrice and allowed to further equilibrate [12, 14, 17].

#### Videomorphometry

Bronchial contractility was evaluated by a stereo microscope Zenith SZR-10 and a digital Optikam-B5 managed by OptikaView7 software (Optika Microscopes, Italy). Small airways were allowed to equilibrate and continuously flushed with fresh KH buffer solution until the luminal area was stable. The area in the lumen was measured by the image processing and analysis software ImageJ [18].

### Acetylcholine and cAMP quantification

Bath supernatant, cell culture medium and airway tissues were collected to quantify the release of acetylcholine and the concentrations of cyclic adenosine monophosphate (cAMP) by using enzyme-linked immunosorbent assay (ELISA) kits according to manufacturers’ instructions in triplicate experiments (BioVision, CA, USA; Cells Biolabs, CA, USA).

Briefly, acetylcholine was converted to choline by adding acetylcholinesterase. After that, free choline was oxidised to betaine via the intermediate betaine aldehyde. The reaction generated products which reacted with the choline probe to generate colour, and the absorbance was measured at 570 nm. Standard curves were prepared using cAMP standard, and sample concentrations were then determined. The detection range of this kit was 10 pM to 5 nM cAMP (http://www.biovision.com/manuals/K615.pdf).

For cAMP detection, an anti-rabbit immunoglobulin G (IgG) polyclonal coating antibody was adsorbed onto a microtitre plate, and cAMP competed with peroxidase cAMP tracer for binding to the plate in the presence of rabbit anti-cAMP polyclonal antibody. Following incubation and wash steps, any peroxidase cAMP tracer bound to the plate was detected by addition of substrate solution. The coloured product formed was inversely proportional to the amount of cAMP. The reaction was terminated by addition of acid, and absorbance was measured at 450 nm. Standard curves were prepared using cAMP standard, and sample concentrations were then determined. The detection range of this kit was 1 to 1000 pM/mL cAMP (http://www.cellbiolabs.com/sites/default/files/STA-500-camp-elisa-kit.pdf).

### Study design

#### Study 1: Evaluation of the interaction between glycopyrronium plus indacaterol on the relaxation of human isolated bronchi pre-contracted with acetylcholine and histamine

Following equilibration of the tissues, bronchial rings/slices were submaximally contracted using acetylcholine (2 μM, inducing 70 % of maximal contraction [EC_70_]). After a plateau was reached, semi-logarithmic concentration response curves (CRCs) were constructed for glycopyrronium and/or indacaterol, alone or together, at their isoeffective concentrations. Each CRC was obtained by the cumulative addition of glycopyrronium and/or indacaterol at intervals of 5-15 min to reach a stable level of relaxation before the next dose administration. In the control groups, cumulative concentrations of vehicle were administered and used as a time control. At the end of the experiments, papaverine (100 μM) was added to the bronchial rings to determine the maximal relaxant response achievable for each isolated bronchus. These experiments were conducted in both an isolated organ bath system and a PCLS system in order to evaluate the relaxation associated with the airway smooth muscle strength and due to the increase in intra-luminal bronchial area [6, 15, 19, 20].

Further experiments were carried out in order to assess the pharmacological interaction between glycopyrronium and indacaterol in human isolated bronchi submaximally contracted using histamine (20 μM, [EC_70_]).

#### Study 2: Evaluation of the long-lasting interaction between glycopyrronium plus indacaterol in human isolated bronchi contracted using transmural stimulation

Each isolated organ bath was fitted with two platinum plate electrodes connected to a stimulator 3165 Multiplexing Pulse Booster (Ugo Basile, VA – Italy) and placed alongside the bronchial rings for EFS. Experiments were performed using trains of 10 Hz EFS (biphasic pulse with a constant current of 10 V, 0.5 ms, 10 s) one pulse every 5 min in order to simulate the vagus nerve firing [13, 21]. After the start of the EFS trains, bronchi were treated for 60 min with glycopyrronium and/or indacaterol, alone or together, at EC_20_. After this step, tissues were washed thrice and the experiment progressed for 12 h. During this time, the bronchial rings were flushed with KH buffer solution at a rate of 30 mL/h. At the end of the experiments, papaverine (100 μM) was added to the bronchial rings to determine the maximal relaxant response achievable for each isolated bronchus [6, 15].

#### Study 3: Influence of glycopyrronium and indacaterol on the release of acetylcholine and cAMP in human isolated bronchi and airway epithelial cells

Human isolated bronchi and airway epithelial cells were submaximally stimulated with carbachol at EC_70_ and treated for 30 min with glycopyrronium and/or indacaterol, alone or together, at EC_30_ (glycopyrronium: 2.0 nM, indacaterol: 5.8 nM). After that, the tissues and supernatants were collected to measure the concentration of acetylcholine and cAMP. The release of acetylcholine has been quantified also in isolated airways treated with histamine (20 μM), in the presence or absence of glycopyrronium and/or indacaterol, alone or together, at EC_30_ (glycopyrronium: 2.02 μM, indacaterol: 4.30 μM). Experiments were also repeated in epithelium-denuded bronchi. In some experiments, human bronchi were pre-treated for 30 min with iberiotoxin (IbTX, 100 nM) [22–25] and the tetanus toxin (TeTX, 10 nM) [26–29] in order to block the KCa^++^ channels and inhibit the synaptic vesicle exocytosis of acetylcholine, respectively. In further experiments, primary human bronchial epithelial cells were pre-treated for 30 min with the organic cation transporter (OCT) inhibitor quinine (100 μM) in order to reduce the release of endogenous acetylcholine from epithelial cells [14].

#### Study 4: Role of cAMP-dependent pathway in glycopyrronium/indacaterol interaction

The role of the cAMP-dependent pathway in modulating the glycopyrronium/indacaterol interaction has been assessed by treating for 30 min human isolated airways, previously submaximally contracted by carbachol at EC_70_, with glycopyrronium and/or indacaterol and/or the activator of the catalytic subunit of adenylate cyclase (AC) forskolin, alone or together in double and triple combinations administered at EC_30_ (forskolin: 56.2 nM; glycopyrronium and indacaterol: concentrations reported in study 3) [30]. Some experiments were carried out also in epithelium-denuded bronchi. The relaxant response was recorded and tissues collected to quantify the concentration of cAMP. Papaverine (100 μM) was used to determine the maximal relaxant response and identify the maximal production of cAMP achievable in human isolated bronchi.

### Analysis

#### Airways tone, acetylcholine release and cAMP concentrations

The contractile relaxation of isolated bronchial rings/slices is expressed as a percentage of the maximal relaxation (E_max_, strength/luminal area) induced by papaverine (100 μM) on the acetylcholine EC_70_ plateau. Appropriate curve-fitting to a sigmoidal model was used to calculate the effect (E), the maximal response (E_max_) and the dose inducing 50 % and 70 % maximal effect (EC_50_ and EC_70_, respectively). The equation used was: response (variable slope) expressed as Y = Bottom + (Top − Bottom)/{1 + 10^[(LogEC_50_ − X)*HillSlope]}, and the pEC_50_ value (pEC_50_ = -LogEC_50_) was used for statistical analysis of the potency [31].

The contractile response to EFS is expressed as a percentage of the effect induced by control EFS preceding the treatment with glycopyrronium or indacaterol. Polynomial curves were constructed by fitting models of biological data using nonlinear regression. Maximal reduction of the EFS contractile tension (E_max_ relaxation) and the onset of action (t_1/2_, min, indicating the time to evoke a half of maximal relaxation) were identified. For every seven bronchial rings mounted in the isolated organ bath system, one was used as a time control [13, 31, 32].

The release of acetylcholine and the concentrations of cAMP were normalised to the wet weight of the isolated airways and expressed as a ratio of the control samples.

#### Interaction analysis

The pharmacological interaction between glycopyrronium and indacaterol in human isolated bronchi was assessed using the Bliss Independence (BI) theory, one of the most commonly used models to study combined effects of substances *in vivo* and *in vitro*. The main assumption of the BI theory is that two or more agents act independently of one another. In particular, if the criterion is fulfilled, the mode, and possibly also the site of action of the compounds in the mixture, always differ. The BI theory for two and three agents are expressed by the following equation, respectively: E(x,y) = Ex + Ey − (Ex*Ey) and E(x,y,z) = Ex + Ey + Ez-(Ex*Ey)-(Ex*Ez)-(Ey*Ez)-(Ex*Ey*Ez), where E is the fractional effect, and x, y and z are the doses of the compounds in a combination experiment. If the combination effect is higher than the expected value from the above equations, the interaction is synergistic, while if this effect is lower, the interaction is antagonistic. Otherwise, the effect is additive and there is no interaction [6, 7, 9, 15, 17, 33]. In this protocol, the BI equation was characterised by x = glycopyrronium and y = indacaterol in both studies 1 and 2, and z = forskolin was included in study 4. The BI approach was used to establish the expected relaxant effect and the expected increase of cAMP induced by the interaction between the investigated drugs at their isoeffective concentrations.

#### Head-to-head comparison

Previous published data on further LABA/LAMA combination (aclidinium/formoterol combination) are currently available [6]. Considering that those results [6] have been produced by our research group by performing *ex vivo* assays undergoing experimental condition identical to that of Study 2, as reported in the above described “Study design” section, we have carried out a head-to-head comparison of glycopyrronium/indacaterol vs. aclidinium/formoterol combinations administered at low concentrations inducing EC_20_, with regard of the pharmacological interaction elicited in course of EFS lasting 12 h. The extent of synergistic interaction expressed as area under the curve at different time intervals (AUC_0-t_), onset of action, E_max_ at plateau and duration of synergistic interaction have been analyzed.

#### Statistical evaluation

Values are presented as mean ± SEM of *n* = 3 bronchi from different subjects. The statistical significance was assessed by the t-test and one-way analysis of variance (ANOVA), and the level of statistical significance was defined as *P* < 0.05. All data analysis was performed using computer software (GraphPad Prism 5, CA, USA) installed on an iMac computer.

### Drugs

The test compounds are highly hygroscopic and were stored in a dry place. The solutions were prepared fresh every day. The products used in this study were obtained from the following sources, and stock solutions were prepared as indicated below: acetylcholine (Sigma-Aldrich, Italy), carbachol (Sigma-Aldrich, Italy), histamine (Sigma-Aldrich, Italy), IbTX (Sigma-Aldrich, Italy), indomethacin (Sigma-Aldrich, Italy), glycopyrronium bromide (NVA237, Novartis), forskolin (Sigma-Aldrich, Italy), papaverine (Sigma-Aldrich, Italy), indacaterol fumarate (QAB149, Novartis), quinine (Sigma-Aldrich, Italy) and TeTX (Sigma-Aldrich, Italy).

Acetylcholine, carbachol, histamine and papaverine were dissolved in distilled water; glycopyrronium, indacaterol and forskolin were dissolved in dimethylsulfoxide (DMSO); indomethacin was dissolved in ethanol and then diluted in KH buffer solution. The maximal amount of ethanol (0.02 %) did not influence isolated tissue response [7, 13–15, 17, 19, 33]. Compounds were stored in small aliquots at −80 °C until their use.

## Results

### Study 1

#### Isolated segmental bronchi

**Fig. 1:**
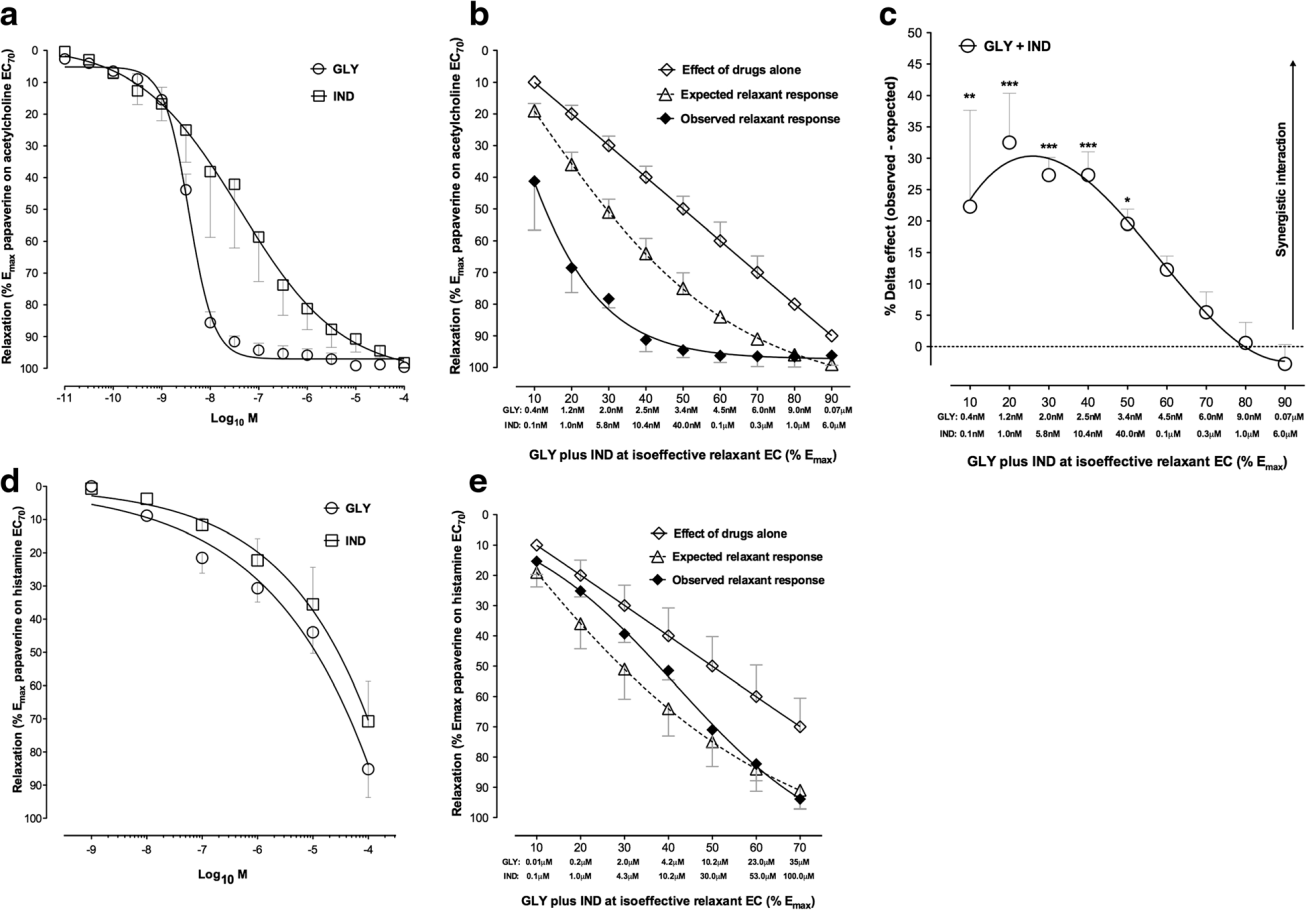
**a** Relaxant effect of glycopyrronium and indacaterol in human isolated bronchi submaximally contracted (EC_70_) with acetylcholine (**a**) and histamine (**d**). Expected and observed relaxant response induced by glycopyrronium and indacaterol in human isolated bronchi submaximally pre-contracted (EC_70_) with acetylcholine (**b**) and histamine (**e**), as predicted by the Bliss Independence theory for the whole range of EC (% E_max_). The isoeffective concentrations of glycopyrronium and indacaterol have been indicated below the X-axis. **c** Delta effect between observed and expected relaxant response induced by glycopyrronium and indacaterol in human isolated bronchi submaximally pre-contracted with acetylcholine (EC_70_), as predicted by the Bliss Independence theory for the whole range of EC (% E_max_). The isoeffective concentrations of glycopyrronium and indacaterol have been indicated below the X-axis. Data are expressed as mean ± SEM from experiments performed using samples from *n* = 3 different subjects. *P < 0.05, **P < 0.01 and ***P < 0.001 vs. expected relaxant response (statistical significance assessed by t test analysis). EC_n_: dose inducing n% maximal effect; E_max_: maximal effect; GLY: glycopyrronium bromide; IND: indacaterol fumarate

Both glycopyrronium and indacaterol induced potent concentration-dependent relaxation of human isolated bronchi submaximally pre-contracted with acetylcholine (−Log dose concentration inducing 50 % maximal effect [pEC_50_] glycopyrronium: 8.44 ± 0.02; pEC_50_ indacaterol: 7.39 ± 0.29); however, glycopyrronium was more potent than indacaterol (*P* < 0.05). Both drugs completely abolished the contractile tone induced by acetylcholine EC_70_ (maximal effect [E_max_] glycopyrronium: 99.68 % ± 0.32 %; E_max_ indacaterol: 92.28 % ± 0.26 %) (Fig. 1a). No significant modification in bronchial tone was noted with the control vehicle used in the experiments (*P* > 0.05).

Glycopyrronium plus indacaterol induced a synergistic relaxant response in human isolated bronchi submaximally pre-contracted with acetylcholine compared to the expected response predicted by the BI theory (Fig. 1b). In particular, the interaction between glycopyrronium and indacaterol was significantly synergistic (*P* < 0.05) at low concentrations (glycopyrronium: 0.4-3.4 nM, indacaterol: 0.1-40.0 nM) and induced a higher relaxant response of +32.51 % ± 7.86 % than the expected additive effect predicted by the BI theory (Fig. 1c).

Glycopyrronium and indacaterol induced similar concentration-dependent relaxation of human isolated bronchi submaximally pre-contracted with histamine (pEC_50_ glycopyrronium: 4.99 ± 0.15; pEC_50_ indacaterol: 4.52 ± 0.18), although both drugs did not completely abolish the contractile tone induced by histamine EC_70_ (E_max_ glycopyrronium: 85.20 % ± 8.58 %; E_max_ indacaterol: 70.76 % ± 11.98 %) (Fig. 1d). Glycopyrronium plus indacaterol elicited additive effect in human isolated bronchi submaximally pre-contracted with histamine, when compared with the expected response predicted by the BI theory (Fig. 1e) (*P* > 0.05 vs. expected relaxant effect).

#### Small airways using PCLS

**Fig. 2:**
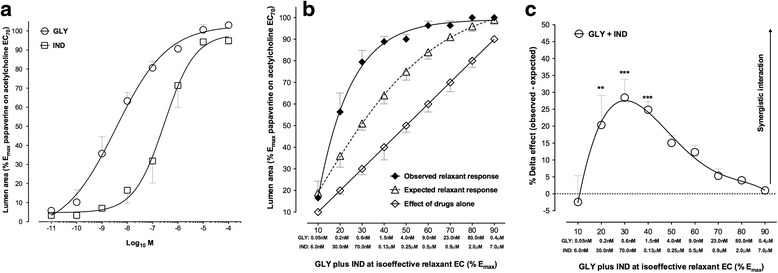
**a** Effect of glycopyrronium and indacaterol on the luminal area of human small airways using PCLS submaximally contracted with acetylcholine (EC_70_). **b** Expected and observed relaxant response induced by glycopyrronium and indacaterol in human small airways using PCLS submaximally pre-contracted with acetylcholine (EC_70_), as predicted by the Bliss Independence theory for the whole range of EC (% E_max_). The isoeffective concentrations of glycopyrronium and indacaterol have been indicated below the X-axis. **c** Delta effect between observed and expected relaxant response induced by glycopyrronium and indacaterol in human small airways using PCLS submaximally pre-contracted with acetylcholine (EC_70_), as predicted by the Bliss Independence theory for the whole range of EC (% E_max_). The isoeffective concentrations of glycopyrronium and indacaterol have been indicated below the X-axis. Data are expressed as mean ± SEM from experiments performed using samples from *n* = 3 different subjects. **P < 0.01 and ***P < 0.001 vs. expected relaxant response (statistical significance assessed by t test analysis). EC_n_: dose inducing n% maximal effect; E_max_: maximal effect; GLY: glycopyrronium bromide; IND: indacaterol fumarate; PCLS: precision cut lung slice

Both glycopyrronium and indacaterol induced potent concentration-dependent relaxation of human PCLSs submaximally pre-contracted with acetylcholine (pEC_50_ glycopyrronium: 8.45 ± 0.23; pEC_50_ indacaterol: 6.53 ± 0.18); however, glycopyrronium was more potent than indacaterol (*P* < 0.01). Both drugs completely abolished the bronchial contraction induced by acetylcholine EC_70_ (E_max_ glycopyrronium: 103.01 % ± 1.59 %; E_max_ indacaterol: 94.85 % ± 1.70 %; *P* < 0.05) (Fig. 2a). No significant modification in bronchial tone was noted with the control vehicle used in the experiments (*P* > 0.05).

Glycopyrronium and indacaterol induced a synergistic relaxant response in human PCLSs submaximally pre-contracted with acetylcholine compared to the expected response predicted by the BI theory (Fig. 2b). In particular, the interaction between glycopyrronium and indacaterol was significantly (*P* < 0.01) synergistic at low concentrations (glycopyrronium: 0.2-1.5 nM, indacaterol: 0.03-0.13 μM) and induced an increased relaxant response of +28.46 % ± 5.35 % compared to the expected additive response predicted by the BI theory (Fig. 2c).

### Study 2

**Fig. 3:**
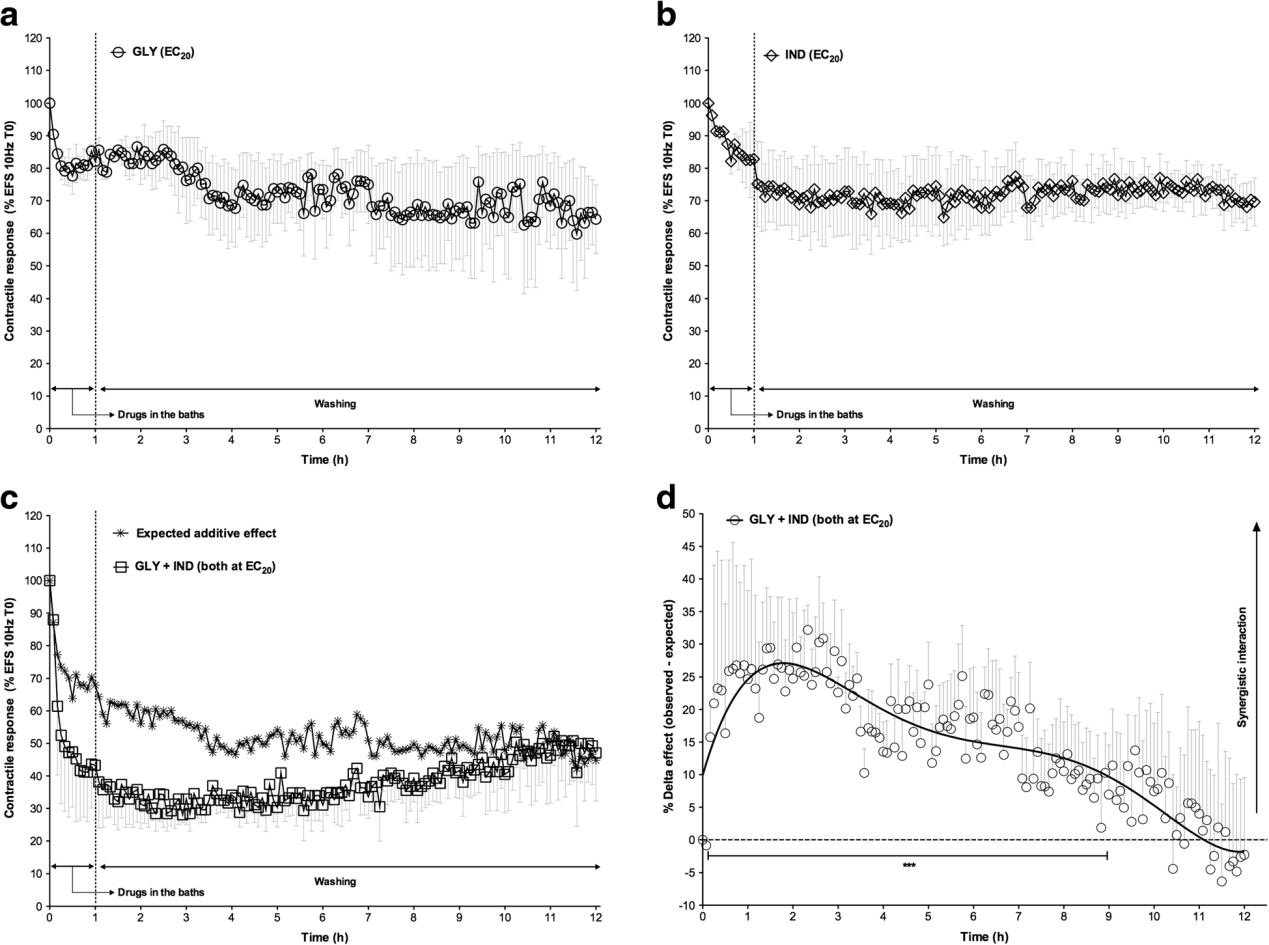
Duration of action for glycopyrronium (**a**) and indacaterol (**b**) in human isolated bronchi stimulated using EFS at 10 Hz to mimic the vagus nerve firing. The observed and expected additive relaxant effect of the drug mixture (both at EC_20_, as predicted by the Bliss Independence theory) are reported during the 12 h of the study (**c**). Delta effect between observed and expected relaxant response, as predicted by the Bliss Independence theory, induced by glycopyrronium and indacaterol in human isolated bronchi contracted using EFS at 10 Hz to mimic the vagus nerve firing during 12 h of the study (**d**). Data are expressed as mean ± SEM from experiments performed using samples from *n* = 3 different subjects. ***P < 0.001 vs. expected relaxant response (statistical significance assessed by two-way ANOVA). EC_n_: dose inducing n% maximal effect; EFS: electrical field stimulation; GLY: glycopyrronium bromide; IND: indacaterol fumarate

Low concentrations of glycopyrronium and indacaterol (0.3 and 30 nM, respectively) yielded an approximately 20 % relaxant response on the bronchial contractile tone induced by EFS 10 Hz during the first hour of the experiment (Fig. 3a and b). In the presence of study drugs in the bath, and before the wash time, both drugs administered alone were unable to elicit a 50 % reduction in the contractile response to EFS. On the contrary, the isoeffective mixture (EC_20_) of low concentrations of glycopyrronium and indacaterol produced a maximal relaxation of 58.82 % ± 15.32 % in the presence of the drugs, and this relaxant effect increased up to 71.95 % ± 2.37 % at 175 min of the experiment, as measured during the wash time (in the absence of drugs). The onset of action for the mixture of drugs was 18.30 ± 9.30 min, and the relaxant effect remained stable for up to 12 h of the experiment (51.76 % ± 13.57 %). The observed relaxant effect, induced by the drug mixture, was considerably higher when compared to the expected relaxant response during the first 9 h of the experiment (Fig. 3c).

The BI interaction analysis shows that low concentrations of glycopyrronium and indacaterol produced a significant (*P* < 0.001) synergistic relaxant effect on the transmural stimulation of human isolated bronchi for 9 h after treatment. The maximal relaxant response was +32.18 % ± 5.44 % higher compared to the expected additive response predicted by the BI theory, and was reached by 140 min of the study (Fig. 3d).

### Study 3

**Fig. 4:**
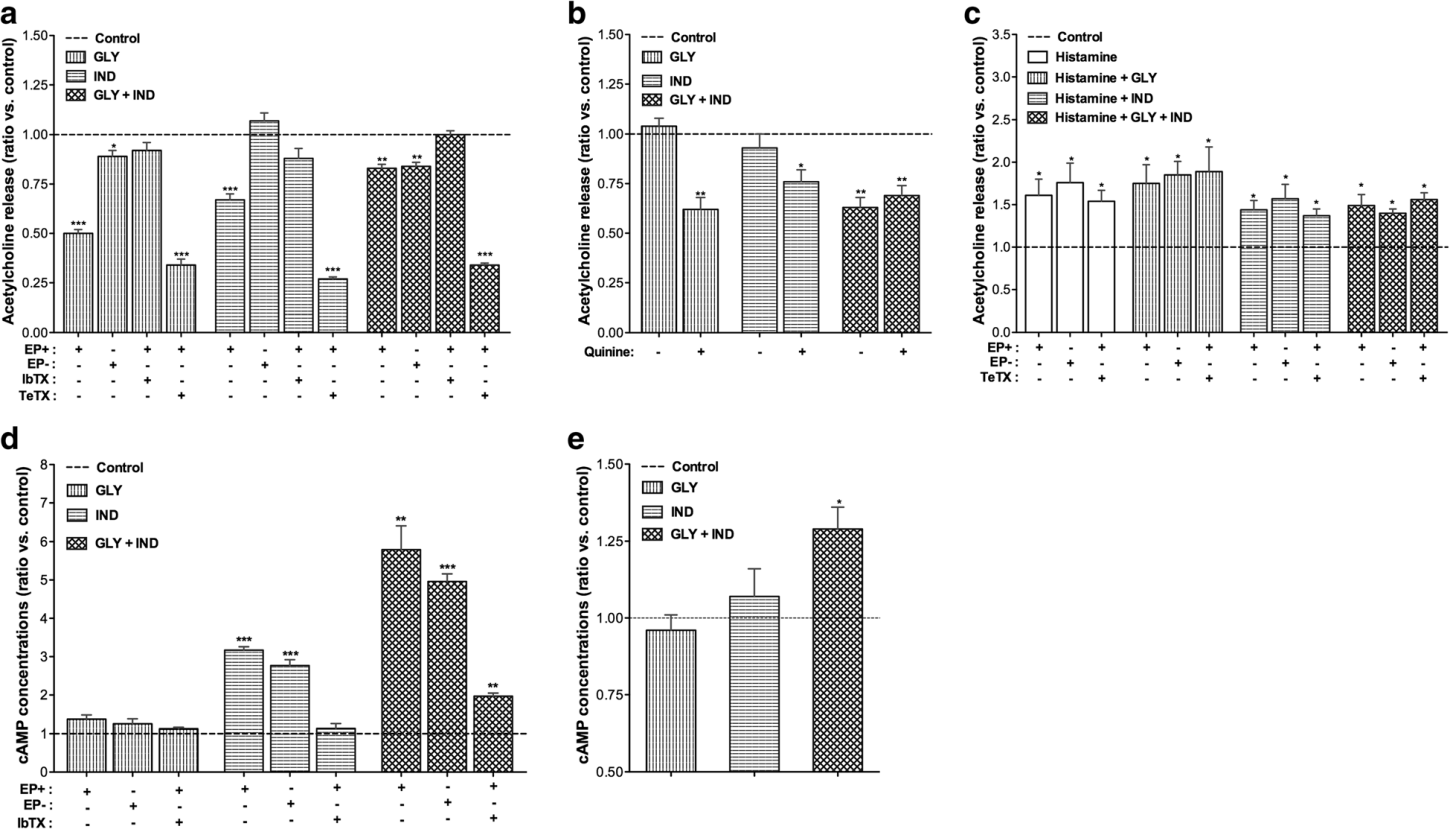
**a** Effect of low concentrations (EC_30_) of glycopyrronium and indacaterol on the release of acetylcholine from intact and denuded epithelium bronchi and the influence of iberiotoxin (100 nM) and tetanus toxin (10 nM). **b** Effect of low concentrations (EC_30_) of glycopyrronium and indacaterol on the release of acetylcholine from primary bronchial epithelial cells submaximally stimulated with carbachol (EC_70_), and influence of quinine (100 μM). **c** Effect of low concentrations (EC_30_) of glycopyrronium and indacaterol on the histamine-induced release of acetylcholine from intact and denuded epithelium bronchi, and the influence of tetanus toxin (10 nM). **d** Effect of low concentrations (EC_30_) of glycopyrronium and indacaterol on the cAMP concentrations in intact and denuded epithelium bronchi and influence of iberiotoxin (100 nM). **e** Effect of low concentrations (EC_30_) of glycopyrronium and indacaterol on the cAMP concentrations in primary bronchial epithelial cells. Data are expressed as mean ± SEM from experiments performed using samples from *n* = 3 different subjects. *P < 0.05, **P < 0.01 and ***P < 0.001 vs. control (dotted line) (statistical significance assessed by t test analysis). cAMP: cyclic adenosine monophosphate; EC_n_: dose inducing n% maximal effect; EP+: epithelium intact; EP−: epithelium denuded; GLY: glycopyrronium bromide; IbTX: iberiotoxin; IND: indacaterol fumarate; TeTX: tetanus toxin

Glycopyrronium and indacaterol, administered alone or in combination at low concentrations, reduced the release of acetylcholine from human isolated bronchi (glycopyrronium: −50.38 % ± 1.77 %, indacaterol: −32.81 % ± 3.45 %; *P* < 0.001 vs. control; combination, −16.82 % ± 2.32 %; *P* < 0.01 vs. control). Removal of the epithelium resulted in inhibition of the effects of both glycopyrronium and indacaterol (+38.95 % ± 2.81 % and +39.90 % ± 4.15 %, respectively; *P* < 0.01 vs. epithelium intact bronchi) but did not lead to modification of the acetylcholine concentrations in the supernatant when the drugs were administered in combination (*P* > 0.05 vs. epithelium intact bronchi). Inhibition of KCa^++^ channels by IbTx completely abolished the effect of glycopyrronium and indacaterol, administered alone or in combination, on the acetylcholine release (*P* > 0.05 vs. control). The blockade of synaptic vesicle exocytosis by TeTX inhibited the release of acetylcholine (−59.99 % ± 1.19 %, *P* < 0.001 vs. control), and both glycopyrronium and indacaterol further enhanced this effect (overall, −68.38 % ± 1.79 %, *P* < 0.001 vs. control) (Fig. 4a).

No modification of acetylcholine release from primary human bronchial epithelial cells was observed at low concentrations of glycopyrronium and indacaterol (*P* > 0.05 vs. control), whereas the glycopyrronium/indacaterol combination significantly reduced the acetylcholine concentrations in the culture medium (−36.63 % ± 4.69 %, *P* < 0.01 vs. control). While the OCT inhibitor quinine inhibited the release of endogenous acetylcholine from epithelial cells (−37.13 % ± 3.29 %, *P* < 0.01 vs. control) in the presence of glycopyrronium and indacaterol (−38.28 % ± 5.73 % and −24.33 % ± 5.65 %, respectively; *P* < 0.05 vs. control), it did not influence (*P* > 0.05) the effectiveness of the glycopyrronium/indacaterol combination (−31.26 % ± 5.24 %, *P* < 0.01 vs. control) (Fig. 4b).

Histamine significantly increased the release of acetylcholine (+61.12 % ± 19.25 %, *P* < 0.05 vs. untreated airways), whereas neither glycopyrronium nor indacaterol administered alone and in combination significantly reduced the histamine-induced acetylcholine release (-4.99 % ± 15.33 % vs. histamine-stimulated airways). Neither epithelium nor TeTX significantly modified the release of acetylcholine mediated by histamine, also in the presence of glycopyrronium and/or indacaterol (*P* > 0.05 vs. epithelium intact airways and TeTX untreated airways) (Fig. 4c).

Although the cAMP concentrations in the isolated bronchi were significantly enhanced with indacaterol (+167.60 % ± 2.33 %, *P* < 0.001 vs. control) but not with glycopyrronium (*P* > 0.05 vs. control), combining low concentrations of glycopyrronium plus indacaterol induced a noteworthy increase in cAMP concentrations (+479.44 % ± 62.40 %, *P* < 0.01 vs. control). In epithelium-denuded bronchi, the modulation of cAMP by glycopyrronium and indacaterol was not different compared with that observed in epithelium intact bronchi (*P* > 0.05). Pre-treatment with IbTX inhibited the effects of indacaterol and the glycopyrronium/indacaterol combination on the increase of cAMP concentrations (*P* < 0.01 vs. IbTX untreated) (Fig. 4d).

Neither glycopyrronium nor indacaterol modified the cAMP concentrations of primary human bronchial epithelial cells (*P* > 0.05 vs. control), whereas the combination of low concentrations of glycopyrronium plus indacaterol induced a significant increase in cAMP concentrations (+29.08 % ± 7.05 %, *P* < 0.05) compared with the control (Fig. 4e).

### Study 4

**Fig. 5:**
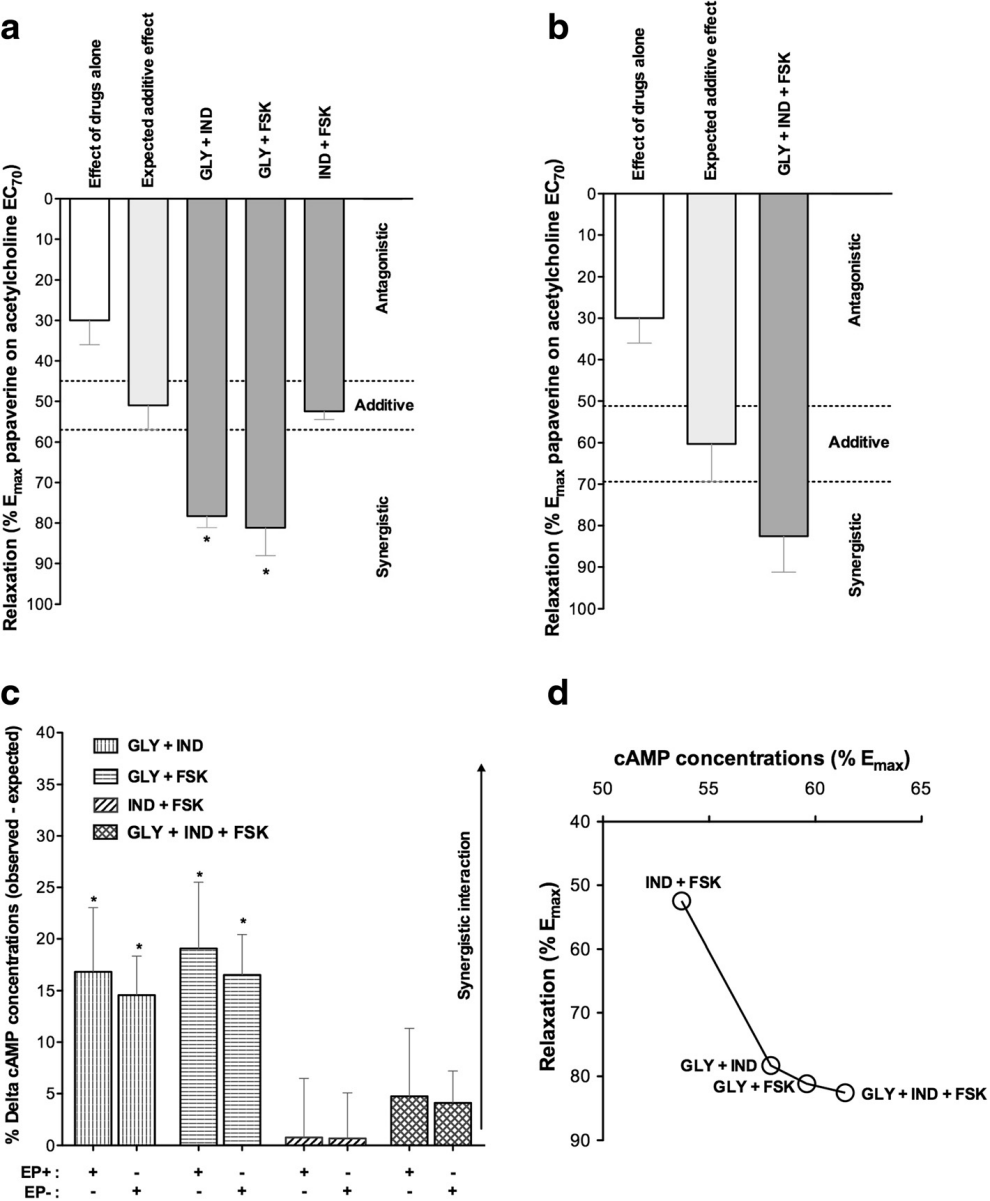
Expected and observed relaxant response induced by low concentrations (EC_30_) of glycopyrronium, indacaterol and forskolin administered as double (**a**) or triple (**b**) combinations in human isolated bronchi submaximally pre-contracted with acetylcholine (EC_70_), as predicted by the Bliss Independence theory. **c** Influence of low concentrations (EC_30_) of glycopyrronium, indacaterol and forskolin administered as double and triple combinations on the delta effect between observed and expected cAMP concentrations in human isolated bronchi submaximally pre-contracted with acetylcholine (EC_70_), as predicted by the Bliss Independence theory. **d** Correlation between the cAMP concentrations induced by glycopyrronium, indacaterol and forskolin combinations and relaxant response of human isolated airways submaximally pre-contracted with acetylcholine (EC_70_). Data are expressed as mean ± SEM from experiments performed using samples from *n* = 3 different subjects. *P < 0.05 vs. expected additive effect as predicted by Bliss Independence equations (statistical significance assessed by t test analysis). cAMP: cyclic adenosine monophosphate; EC_70_: dose inducing 70 % maximal effect; E_max_: maximal effect; EP+: epithelium intact; EP−: epithelium denuded; FSK: forskolin; GLY: glycopyrronium bromide; IND: indacaterol fumarate

Low concentrations of glycopyrronium/indacaterol and glycopyrronium/forskolin combinations inducing alone EC_30_ synergistically increased the relaxant response of isolated airways submaximally pre-contracted with acetylcholine (+27.32 % ± 2.83 % and +30.19 %% ± 6.87 %, respectively; *P* < 0.05 vs. expected additive effect), whereas the indacaterol/forskolin combination elicited only an additive effect when compared with the expected relaxation predicted by the BI theory (Fig. 5a). Although the triple combination glycopyrronium/indacaterol/forskolin produced a greater broncholitic effect than that expected by the BI theory, (+22.29 % ± 8.67 %), the extent of this effect was not significant when compared with the additive effect as predicted by the BI theory (*P* > 0.05) (Fig. 5b).

The glycopyrronium/indacaterol and glycopyrronium/forskolin combinations both synergistically increased the concentrations of cAMP (overall, +17.98 % ± 6.29 %, *P* < 0.05 vs. expected additive effect), whereas the indacaterol/indacaterol and triple glycopyrronium/indacaterol/forskolin combinations induced only additive effects, when compared with the expected effect as predicted by the BI theory (*P* > 0.05). The presence of epithelium did not modulate the interaction characteristics for all drugs combinations (*P* > 0.05 vs. epithelium-denuded bronchi) (Fig. 5c).

The increase of cAMP elicited by the double and triple drugs combinations was significantly correlated with the extent of bronchial relaxant response (Pearson’s r 0.95, R^2^ 0.90; *P* < 0.05) (Fig. 5d).

### Head-to-head comparison

The head-to-head comparison of glycopyrronium/indacaterol vs. aclidinium/formoterol combinations indicated that the overall extent of synergistic interaction (AUC_0-12_) and onset of action (T_1/2_) were similar for both these drugs combinations (*P* > 0.05). Synergism AUC_0-3_ and AUC_0-6_ induced by aclidinium/formoterol combination was significantly greater that that elicited by glycopyrronium/indacaterol combination (+41.70 ± 13.49, *P* < 0.01), as confirmed by the difference in E_max_ at plateau (+17.01 % ± 7.56 %, *P* < 0.01). On the other hand, the analysis of the duration of synergism indicated significant superiority of glycopyrronium/indacaterol combination vs. aclidinium/formoterol combination (+3 h, *P* < 0.05). Detailed comparison results are reported in Table 2.

**Table 2:** Comparison of the synergism elicited by glycopyrronium/indacaterol vs. aclidinium/formoterol combinations in human isolated bronchi stimulated by EFS at 10 Hz

	Glycopyrronium/indacaterol	Aclidinium/formoterol	*P*
AUC			
0-1 h	20.22 ± 8.38	27.39 ± 3.51	NS
0-3 h	72.65 ± 18.72	108.25 ± 10.12	**
0-6 h	127.26 ± 28.81	175.07 ± 16.86	**
0-9 h	164.64 ± 41.88	186.40 ± 29.87	NS
0-12 h	175.43 ± 61.55	159.25 ± 53.19	NS
Onset (T_1/2_, min)	18.30 ± 9.30	15.5 ± 3.5	NS
E_max_ (plateau, %)	25.93 ± 9.41	42.94 ± 7.56	***
Duration of synergism (h)	9:00 ± 0:11	6:00 ± 0:12	***

AUC_(0-t)_: area under the curve for specific time intervals; EFS: electrical field stimulation; NS: not significant (P > 0.05). Data are expressed as mean ± SEM from experiments performed using samples from *n* = 3 different subjects. **P < 0.01 and ***P < 0.001 (statistical significance assessed by two-way ANOVA)

## Discussion

The results of this study demonstrate that both glycopyrronium and indacaterol have the ability to induce potent, significant and long-lasting relaxation of both medium and small human isolated bronchi pre-contracted with acetylcholine. The co-administration of glycopyrronium and indacaterol produces a synergistic inhibition of ASM tone via modulating the cAMP-dependent pathway, especially when these drugs are administered at low concentrations. Intriguingly, when glycopyrronium and indacateriol were administered at low concentrations in our experimental setting, their ratio was consistent with that of the currently approved fixed dose combinations (FDCs), namely 15.6/27.5 μg in United States and 50/110 μg in European Union [34, 35].

Overall, the functional data described in this study are consistent with those concerning the pharmacological characterisation of the interaction between aclidinium bromide and formoterol fumarate in human isolated bronchi [6]. Nevertheless, the glycopyrronium/indacaterol combination produced a greater synergistic interaction in both medium bronchi and bronchioles when compared with that induced by the aclidinium bromide/formoterol fumarate combination. In addition, we must highlight that while formoterol fumarate administered alone did not completely relax small airways, indacaterol was able to abolish the contractile tone of PCLS preparations [6]. As expected, the duration of the synergistic effect of glycopyrronium plus indacaterol was markedly longer than that elicited by combining aclidinium bromide with formoterol fumarate. In fact, a significant synergism between glycopyrronium and indacaterol was detectable for at least 9 h. On the contrary, combining aclidinium bromide with formoterol fumarate induced a greater post-administration bronchorelaxant peak, though the synergism was significant for only 6 h after the administration of the two drugs [6]. In any case, the head-to-head comparison of glycopyrronium/indacaterol vs. aclidinium/formoterol combinations indicated that the overall extent of synergistic interaction and onset of action were similar for both these LABA/LAMA combinations, at least undergoing the experimental conditions set up in our *ex vivo* model of human isolated bronchi. In fact, we must consider that a recent synthesis of the currently available clinical data suggested for a rank of effectiveness among the approved doses of LAMA/LABA FDCs, with glycopyrronium/indacaterol (15.6/27.5 mg and 50/110 mg) eliciting greater FEV_1_ increase than aclidinium/formoterol (400/12 mg) in COPD patients, when compared with the respective monocomponents [36]. However, this discrepancy may be related with the fact that glycopyrronium/indacaterol and aclidinium/formoterol combinations, administered at the currently approved doses, may be not delivered into the lung at isoeffective concentrations [36].

When one agent interacts with its specific G protein-coupled receptor (GPCR), the effect of another agent on its GPCR may change, leading to a possible pharmacological interaction [37, 38]. Since our study has provided evidences for a synergistic cross-talk between an anti-muscarinic agent and a β_2_-adrenoceptor agonist undergoing cholinergic stimulation, we have investigated whether the inhibition of muscarinic GPCRs may transmit the signal to β_2_-adrenoceptor GPCR also in course of histamine-induced bronchoconstriction. The bronchial contractile tone induced by histamine is mediated by both direct activation of histaminergic receptors expressed on human ASM, and facilitator effect of the acetylcholine release from parasympathetic nerve terminals [38–40]. The findings of our study represent a significant step forward the study of Aizawa et al. [40], by providing the evidence that the increase in isometric tension elicited by histamine is manly mediated by the resease of acetylcholine via the direct action of histamine on the vagus efferent nerve terminals, independently by neural conduction. In fact, neither TeTX nor epithelium altered the histamine-induced increase of acetylcholine release. Furthermore, neither glycopyrronium nor indacaterol were able to reduce the release of acetylcholine at control levels, even when these drugs were administered in combination. Therefore, the lack of synergism between glycopyrronium and indacaterol on the human ASM contractility histamine-mediated may be explained by the lack of a direct influence of either these drugs on the bronchial histaminergic pathway. Our results confirm that both anti-muscarinic agents and β_2_-adrenoceptor agonists are less potent and effective in reducing the bronchial tone elicited by histamine when compared with their impact on the cholinergic tone [19, 33, 41], and that no cross-talk exists between muscarinic and to β_2_-adrenoceptor GPCRs in course of histaminergic stimulation.

Nevertheless, it has been recently reported that the bronchoprotection by a LABA may be synergistically enhanced by a LAMA, at least *in vivo* in guinea-pigs [42, 43]. Indeed, the results of these studies are in evident contrast with our findings, and probably any discrepancy may be related with the considerable differences between our methodological approach and that performed by Smit and colleagues [42, 43]. However, we must highlight that the data we have presented here may be of great interest, because the responses of autonomic nervous system and ASM are specific for species and for tissues [40], and results obtained from human isolated tissues have certainly a greater translational potential when compared with those obtained from animal models [44].

In this study, we have also attempted to elucidate the mechanism(s) underlying the bronchorelaxant interaction between LABAs and LAMAs.

We assumed that the activation of β_2_-adrenoceptors by indacaterol would have no effect on the release of acetylcholine from parasympathetic nerves, as previously documented by using human isolated trachea stimulated with the β_2_-adrenoceptor agonist isoprenaline [45]. On the contrary, our data showed that indacaterol was able to reduce the release of acetylcholine, a phenomenon that was dependent by the bronchial epithelium and reverted by blocking the IbTX-sensitive KCa^++^ channels.

Since we have confirmed the neuronal origin of acetylcholine by inhibiting its release using TeTX, we can assume that in human bronchi indacaterol has a protective role against the neuronal release of acetylcholine. There is experimental documentation in laboratory animals that β_2_-adrenoceptor stimulation may elicit a paradoxical facilitation of acetylcholine release from isolated trachea via the activation of a cAMP/cAMP-dependent protein kinase cascade [45]. Our data indicate that this is not the case in human bronchial tissue.

Surprisingly, contrary to our assumption that an anti-muscarinic agent would facilitate neurogenic transmission due to inhibition of the pre-synaptic muscarinic M_2_ autoreceptor, glycopyrronium inhibited the parasympathetic release of acetylcholine [9]. So far, it is well known that anti-muscarinic agents are not specifically selective for the post-synaptic muscarinic M_3_ receptor [46]. In fact, LAMAs may bind to human muscarinic M_1_-M_5_ receptors in a concentration-dependent manner, although they dissociate more slowly from the muscarinic M_3_ receptor than they do from the others [47]. In particular, glycopyrronium showed no selectivity in its binding to the muscarinic M_1_-M_3_ receptors [48]. However, a 3-5–fold higher affinity was observed for the muscarinic M_3_ receptor compared to the muscarinic M_1_ and M_2_ receptors, and the Schild plot analysis demonstrated that glycopyrronium has a higher affinity for muscarinic M_1_ and M_3_ receptors compared to the muscarinic M_2_ autoreceptor [48]. Thus, at the level of post-ganglionic parasympathetic neurons, glycopyrronium prevalently inhibits the muscarinic M_1_ receptor expressed on the body cell compared with the inhibition elicited on the muscarinic M_2_ autoreceptor localised on the post-ganglionic fibre [49]. Since the muscarinic M_1_ receptor is facilitatory to nicotinic receptors and is involved in setting the efficacy of ganglionic transmission [49, 50], the overall inhibitory effect of glycopyrronium leads to reduced parasympathetic transmission.

Analogous to indacaterol, modulation of the acetylcholine release by glycopyrronium was related with the integrity of the bronchial epithelium and the functionality of IbTX-sensitive KCa^++^ channels. Unfortunately, we did not detect any synergistic effect on the inhibitory release of parasympathetic acetylcholine when low concentrations of indacaterol and glycopyrronium were administered in combination.

The autonomic control of human ASM tone is primarily mediated by the release of acetylcholine from parasympathetic fibers [51]. However, a non-neuronal cholinergic system exists at the level human airways [52]. The synthesis, recycling, storage and release of non-neuronal acetylcholine is mediated by several mechanisms such as choline acetyltransferase (ChAT), ChAT-like enzymes, carnitine acetyltransferase (CarAT), high-affinity choline transporter (CHT1), vesicular acetylcholine transporter (VAChT) and OCT [53, 54]. Thus, in addition to the parasympathetic release of acetylcholine, this transmitter may have a crucial local auto-/paracrine role in regulating several aspects on the innate mucosal defense mechanisms, including mucociliary clearance, regulation of macrophage function and modulation of sensory nerves [55].

Since our findings showed that removal of the bronchial epithelium may influence the release of acetylcholine from human bronchi, we further investigated the role of indacaterol and glycopyrronium on the bronchial non-neuronal cholinergic system [52, 55, 56]. Interestingly, although indacaterol and glycopyrronium alone did not modify the release of acetylcholine from primary human bronchial epithelial cells, a combination of these drugs inhibited the epithelial release of acetylcholine with the same extent to the inhibitory effect induced by the OCT inhibitor quinine.

Taken together, these evidences allow us ruling out a direct influence of synaptic postganglionic nerve endings in the synergistic interaction between glycopyrronium and indacaterol. Nevertheless, epithelium may be at least partially responsible for the synergistic effect of glycopyrronium/indacaterol, since the drug combination was effective in reducing endogenous and non-neurogenic release of acetylcholine from bronchial epithelial cells compared with either drug administered alone. This latter evidence may explain, to some extent, the relevant synergistic interaction between glycopyrronium and indacaterol in PCLS preparations, since at the level of human bronchioles the density of vagal innervation is insignificant or even absent, thus suggesting a role of the non-neuronal cholinergic system [57, 58]. In fact, in peripheral airways, the muscarinic M_3_ receptor is expressed and may be activated by acetylcholine released from epithelial cells that may express ChAT in response to inflammatory stimuli [59].

In any case, the function of muscarinic receptors localized on human bronchial epithelium is still speculative. The activation of muscarinic M_1_ may induce proliferation of isolated tracheal epithelial cells, and bronchial epithelial muscarinic M_3_ receptor seems to mediate the release of diffusible factors, modulating contractility of underlying ASM [60]. Furthermore, the role of muscarinic M_2_ receptor remains unclear in bronchial epithelium [61]. Therefore, although the nature of these factors remains unknown, we cannot exclude that muscarinic antagonists may reverse the G_i_/KCa^++^ channel inhibitory linkage induced by the activation of muscarinic M_2_ receptor at the level of airway epithelial cells, concurring with the similar effect elicited by β_2_-adrenoceptor agonists through intracellular cAMP elevation and reducing the release of acetylcholine [37].

Since we have demonstrated that the synergism between a LABA and a LAMA cannot be adequately explained by the modulation of acetylcholine release, we investigated further mechanisms that might directly engage the ASM. Our data suggest that cAMP elevation induced in ASM by combining low doses of glycopyrronium plus indacaterol seems to be the main cause that explains the synergistic interaction between these bronchodilators. In fact, a noteworthy enhancement of cAMP levels was detected in human isolated bronchi treated with the glycopyrronium/indacaterol combination compared with isolated airways treated with the monocomponents. As expected, the concentrations of cAMP were not modulated by glycopyrronium, whereas indacaterol enhanced the cAMP levels by approximately three fold, independent by the presence of epithelium. On the other hand, the glycopyrronium/indacaterol combination elicited a noteworthy increase in cAMP concentrations up to approximately seven fold.

Furthermore, our results suggest that the activity of IbTX-sensitive KCa^++^ channels is crucial for generating this significant cAMP elevation. Stimulation of the β_2_-adrenoceptor activates the KCa^++^ channels through both cAMP-dependent and -independent mechanisms, leading to hyperpolarisation of cell membrane and, consequently, to ASM relaxation [62, 63]. To the best of our knowledge, we have demonstrated for the first time in human ASM that not only cAMP may influence the activity of KCa^++^ channels, but also that the IbTX-sensitive KCa^++^ channels themselves may modulate cAMP increase following the concomitant activation of the β_2_-adrenoceptor and inhibition of muscarinic receptors.

Several pathways have been proposed to explain the intracellular cross-talk between β_2_-adrenoceptors and muscarinic receptors at the level of the human ASM [9]. In our study, the role of IbTX-sensitive KCa^++^ channels seems to be predominant, since these channels may induce a direct reduction of ASM tone and, equally important, regulate the intracellular concentrations of cAMP [64, 65]. Our data suggest that the functionality of IbTX-sensitive KCa^++^ channels is crucial for allowing the synergistic interaction between a LABA and a LAMA, leading to a sustained and intense bronchorelaxant effect via cAMP elevation.

Since the bronchial epithelium expresses both β_2_-adrenoceptors and muscarinic receptors [59], we also investigated whether a LABA/LAMA combination might have a role in the synthesis of cAMP in primary human bronchial epithelial cells as well. Intriguingly, glycopyrronium and indacaterol administered alone at low concentrations did not modify the basal level of cAMP, whereas the combination of these drugs increased the cAMP levels in bronchial epithelial cells. This finding suggests that the bronchial epithelium may also contribute to the modulation of cAMP levels in human bronchi following the stimulation of β_2_-adrenoceptors and inhibition of muscarinic receptors, thus supporting the synergistic interaction elicited by the glycopyrronium/indacaterol combination.

The findings of this study prove that the synergistic interaction between glycopyrronium and indacaterol is both directly and indirectly mediated downstream by the stimulation of the cAMP-dependent pathway. In fact the block of muscarinic receptors by glycopyrronium induced synergism in the presence of cAMP stimulant agents, such as indaceterol or forskolin. On the other hand, modulating the same intracellular pathway, by combining indacaterol with forskolin, produced only an additive relaxant response.

Our effort to characterize the interaction between different bronchorelaxant agents has demonstrated that synergism may be elicited only when the pharmacological interventions are focused on specific different pathways that, unexpectedly, converge in a noteworthy downstream modulation of intracellular messengers specific for only one of the involved pathways, namely the cAMP increase induced by the activation of β_2_-adrenoceptors. In effect, no synergistic interaction was produced by the direct activation of AC by forskolin and the concomitant stimulation of β_2_-adrenoceptors, even in the presence of an anti-muscarinic agent.

Finally, but not less important, these findings fully corroborate the main assumption of the BI criterion, that if two or more agents act independently of one another, neither one interferes with the other, but each contributes to a common result leading to additive effect. In fact, although β_2_-adrenoceptors agonists and anti-muscarinic agents interact with different and independent smooth muscle cell membrane receptors, they orchestrate downstream intracellular signalling pathways that interfere each other eliciting synergistic bronchorelaxant interaction [9]. Therefore, the addition of a second bronchodilator agent should be assessed considering the interference on the signalling cross-talk between different intracellular pathways.

## Conclusions

In conclusion, the findings of this study suggest that co-administation of glycopyrronium and indacaterol at low concentrations leads to synergistic improvement of bronchodilation in both medium and small airways, when compared with either drug administered alone, due to a significant increase in cAMP concentrations at the level of both ASM and bronchial epithelial cells and a decrease in the release of acetylcholine from the epithelium.

## Abbreviations

BI, Bliss Independence; CRC, concentration response curve; cAMP, cyclic adenosine monophosphate; E, effect; EC, effective dose concentration; EC_n_, dose concentration inducing n% maximal effect; EFS, electrical field stimulation; E_max_, maximal effect; FSK, forskolin; IbTX, iberiotoxin; KCa^++^ channels, calcium activated potassium channels; NVA237, glycopyrronium bromide; OCT, organic cation transporter; PCLS, precision cut lung slice; pEC_50_, −LogEC_50_; QAB149, indacaterol fumarate; TeTX, tetanus toxin.
